# Reciprocal Complementation of the Tumoricidal Effects of Radiation and Natural Killer Cells

**DOI:** 10.1371/journal.pone.0061797

**Published:** 2013-04-25

**Authors:** Kai-Lin Yang, Yu-Shan Wang, Chao-Chun Chang, Su-Chen Huang, Yi-Chun Huang, Mau-Shin Chi, Kwan-Hwa Chi

**Affiliations:** 1 Department of Radiation Therapy and Oncology, Shin Kong Wu Ho-Su Memorial Hospital, Taipei, Taiwan; 2 Institute of Radiation Science and School of Medicine, National Yang-Ming University, Taipei, Taiwan; Technische Universitaet Muenchen, Germany

## Abstract

The tumor microenvironment is a key determinant for radio-responsiveness. Immune cells play an important role in shaping tumor microenvironments; however, there is limited understanding of how natural killer (NK) cells can enhance radiation effects. This study aimed to assess the mechanism of reciprocal complementation of radiation and NK cells on tumor killing. Various tumor cell lines were co-cultured with human primary NK cells or NK cell line (NK-92) for short periods and then exposed to irradiation. Cell proliferation, apoptosis and transwell assays were performed to assess apoptotic efficacy and cell viability. Western blot analysis and immunoprecipitation methods were used to determine XIAP (X-linked inhibitor of apoptosis protein) and Smac (second mitochondria-derived activator of caspase) expression and interaction in tumor cells. Co-culture did not induce apoptosis in tumor cells, but a time- and dose-dependent enhancing effect was found when co-cultured cells were irradiated. A key role for caspase activation via perforin/granzyme B (Grz B) after cell-cell contact was determined, as the primary radiation enhancing effect. The efficacy of NK cell killing was attenuated by upregulation of XIAP to bind caspase-3 in tumor cells to escape apoptosis. Knockdown of XIAP effectively potentiated NK cell-mediated apoptosis. Radiation induced Smac released from mitochondria and neutralized XIAP and therefore increased the NK killing. Our findings suggest NK cells in tumor microenvironment have direct radiosensitization effect through Grz B injection while radiation enhances NK cytotoxicity through triggering Smac release.

## Introduction

Radiation is a highly effective tumoricidal modality, but its efficacy is modulated by the tumor microenvironment [1], [2]. Many clinical studies have shown that the intra-tumoral presence of CD8^+^ cells, NK cells, CD4+ cells, and dendritic cells (DC) is positively correlated with survival, while the presence of macrophages and regulatory T cells predict poor responsiveness to therapy and survival [3], [4], [5]. There is increased interest in modulation of immune cells infiltrating the tumor microenvironment to enhance the therapeutic efficacy of radiation [6], [7].Patients received vaccine before the standard chemotherapy/radiotherapy to achieve a better result has successfully reported on prostate and head and neck cancer [8], [9], [10]. There is evidence that immune-mediated microenvironmental change has occurred during tumor progression and after therapy. The specific T cells were present before radiation and a cascade of antigen release after radiation may further enhance polyclonal response [8], [10]. The combination of immunotherapy and radiotherapy is theoretically synergistic and complementary to each other. Nevertheless, it is not clearly understood why an improved immunological environment is critical for the efficacy of subsequent radiotherapy nor why an irradiated tumor improves the subsequent immunotherapy effect.

The creation of a favorable host anti-tumor immune microenvironment by in situ delivery of interleukin-2 (IL-2) and granulocyte macrophage colony growth factor (GM-CSF) genes into the peri-tumoral site resulted in improved radio-responsiveness and systemic anticancer immunity [11]. Timar et al. reported that peri-tumoral injection of neoadjuvant leukocyte interleukin augmented the tumor sensitivity to subsequent radiation therapy and chemotherapy in oral cancer [12]. We found that neoadjuvant immunotherapy given before radiotherapy improved the radiosensitization effect over immunotherapy given after radiotherapy, through activation of NK cells [13].

We hypothesized that NK cells sensitized target cells to radiotherapy. The most important apoptotic machinery activated by effector-target cell contact is likely caspase, which is initiated by granzyme B (Grz B)/perforin [14]. Various mechanisms contribute to resistance of tumor cells to immune cell killing [15], [16], [17]. In general, the XIAP/Smac pathway is important for full activation of autoprocessing of caspases [18], [19]. The XIAP protein can directly inhibit caspase activity and regulate death receptor-mediated apoptosis induced by immune cells [20]. The inhibitory action of XIAP is counteracted by Smac, a mitochondrial protein that is released into the cytosol during apoptosis, binds to XIAP, and disrupts its activity [21]. Breaking tumor resistance to immune cells by concomitant low-dose radiation has been reported, but the underlying mechanism is poorly understood [22].

We show here that NK cells significantly enhance the radiation effect on target cells without killing them. Caspase activation after radiation was induced in target cells after co-culture with NK cells but not in target cells without co-culture. Immunotherapy alone (co-cultured only) resulted in increased XIAP binding of caspase-3 in the cytosol, thus escaping apoptosis, whereas irradiating co-cultured cells resulted in a re-localization of XIAP into the mitochondria and induced a release of Smac from the mitochondria to inhibit cytosolic XIAP to enhance apoptosis. This finding provides new evidence of reciprocal complementation between the tumoricidal effects of radiotherapy and immunotherapy.

## Materials and Methods

### Cells and Culture Conditions

The effector cells including primary human NK cells (pNK) isolated from Human peripheral mononuclear cells (PBMC) and human NK-92 cell line. The PBMC was provided by the Taipei Blood Center (TBC) following the guidelines of the Institutional Review Board of TBC. The TBC provide the donor bloods who have already signed the consent of donation to research use and our proposal has to be passed their IRB. The target cells including human lung adenocarcinoma cells (A549), nasopharyngeal cancer cell line (CNE-1), cervical cancer cells (HeLa), hepatoma cells (Hep3B) and breast cancer cells (MCF-7) were purchased from American Type Culture Collection (ATCC), and maintained in DMEM (Invitrogen, Verviers, Belgium) containing 10% heat-inactivated fetal bovine serum (FBS), 2 mM L-glutamine, 100 units/mL penicillin, and 100 µg/mL streptomycin (Sigma, St. Louis, MO). The prostate carcinoma cell line PC-3 and colon carcinoma cell line WiDr were purchased from the Culture Collection and Research Center (Hsinchu, Taiwan), and cultured respectively in complete Ham’s F-12 and α-medium (Invitrogen) supplemented with 10% FBS, glutamine, penicillin, and streptomycin. Human NK-92 cell line was purchased from the Culture Collection and Research Center (Hsinchu, Taiwan), and fresh batches were thawed every year. NK-92 cells were propagated in α-medium supplemented with 12.5% heat-inactivated FBS, 12.5% horse serum, 1.5 g/L sodium bicarbonate, 0.2 mM myo-inositol, 0.1 mM 2-mercaptoethanol, 0.02 mM folic acid (Sigma) and 100 units/mL IL-2 (Proleukin, Chiron, Emeryville, CA). Unconjugated anti-FasL (clone 100419; R&D systems) antibody was used for neutralization experiments.

### Primary Human NK Cell Isolation

pNK cells were isolated from PBMC by negative selection using pNK cell Isolation Kit II and MACS columns (Miltenyi Biotech) following the manufacturer’s protocol. Experiments were performed when purity of pNK cells was more than 95% as determined by flow cytometry.

### MTS Cell Proliferation Assay

Various tumor cell lines were cultured at a density of 1.0×10^5^ cells/well in 96-well round-bottom plates (Falcon) containing 200 µl of medium. Tumor cells (1×10^6^) were cultured with or without NK cells (2.5×10^6^) in different combinations for 4 h. After 4 h, the non-adherent NK cells were washed away and tumor cells were exposed to 800 cGy of irradiation. Tumor cells were maintained for 2 days at 37°C in a 5% CO_2_ humidified atmosphere. The proliferation rate of the cells was measured using an MTS assay (CellTiter 96 aqueous one-solution cell proliferation assay; Promega). Forty microliters of CellTiter 96 aqueous one-solution were added to each well. After 4 h of incubation, the UV absorbance of the solution was measured at a wavelength of 490 nm. All MTS assays were done in triplicate.

### Apoptosis Assay

CNE-1 cells and pNK or NK-92 cells were cultured, trypsinized as described above, and washed twice with PBS. Apoptosis was confirmed using an Annexin V Apoptosis Kit (BD Pharmingen) according to the manufacturer’s instructions. Briefly, tumor cells were washed 3 times with PBS; then, some cells were analyzed immediately for apoptosis using Annexin V/PI staining. Washed cells were supplemented with 1% BSA and then stained directly with 10 µL of PI and 2.5 µL Annexin V-FITC, after the addition of 222.5 µL of binding buffer. Immediately after 10 min incubation in the dark on ice, the cells were analyzed by flow cytometry. The percentage of positive cells was determined by using a FACSCalibur cytometer and Cell Quest Pro software (Becton Dickinson, Mountain View, CA).

### Transwell Assay

CNE-1 cells (0.5×10^6^) were placed in the lower Transwell chamber (Corning Glass Works, Corning, NY) and a filter with a 0.8-µm pore size was placed on top of the chamber. Aliquots of NK-92 cells (1.25×10^6^ cells) were applied to the upper chamber, and the chambers were incubated at 37°C in 5% CO_2_ for 4 h. CNE-1 cells were analyzed by Annexin V/PI apoptosis detection kit as described above.

### Western Blot Analysis

For protein analysis, cells were lysed for 5 min at room temperature in a buffer of 150 mM NaCl, 50 mM Tris (pH 8.0), 5 mM EDTA, 1% (v/v) Nonidet p-40, 1 mM phenylmethylsulfonyl fluoride, 20 µg/mL aprotinin, and 25 µg/mL leupeptin (Sigma). Total protein concentration of lysates was measured using the Bio-Rad protein assay reagent. Cell lysates (100 µg) were electrophoresed on a 12% polyacrylamide gel, transferred onto Immobilon-P PVDF membrane (Millipore, Bedford, MA), and blocked in PBS-Tween 20 and 10% nonfat milk for 2 h at room temperature. The filter was incubated with specific antibodies to anti-Fas, anti-caspase-3, -8, or -9 (Santa Cruz Biotechnology, Santa Cruz, CA), anti-XIAP, or anti-Smac antibody (Cell Signaling Technology, Beverly, CA) for 2 h at room temperature in PBS-0.05% Tween 20 containing 5% nonfat milk, followed by 1 h incubation at room temperature with horseradish peroxidase-conjugated secondary antibodies (Jackson ImmunoResearch Laboratories, West Grove, PA) in the same buffer. Blots were developed on X-ray film by using a chemiluminescent detection system (ECL; GE Life Science, Buckinghamshire, UK). The protein bands of caspase 3 and pro-caspase 3 on X-ray film were scanned and densitometrically analysed with ImageJ software (US National Institutes of Health). Results are expressed as the ratio pro-caspase/caspase 3 (a low ratio is indicative of apoptosis) [23].

### RNA Preparation and Real-time PCR

RNA was extracted with Trizol (Invitrogen, Carlsbad, CA) and chloroform, then precipitated with isopropanol, according to the manufacturer’s recommendations. Further purification was obtained with the RNeasy Mini Kit (Qiagen, Valencia, CA). Integrity and purity were verified by spectrophotometry, and the quality was assessed by electrophoresis on agarose gels. Double-stranded cDNA was synthesized from total RNA. Real-time PCR was performed using a LightCycler rapid thermal cycler system (Roche Diagnostics Ltd, Lewes, UK) according to the manufacturer’s instructions. Reactions were performed in a 20-µL volume with 0.5 µM primers and MgCl_2_ concentration optimized between 2–5 mM. Nucleotides, Taq DNA polymerase, and buffer were included in the LightCycler-DNA Master SYBR Green I mix (Roche Diagnostics). A typical protocol took approximately 15 min to complete and included a 30-s denaturation step followed by 45 cycles with denaturation at 95°C for 10 s, annealing at 55°C for 5 s, and extension at 72°C for 10 s. To confirm amplification specificity, the PCR products from each primer pair were subjected to melting curve analysis and subsequent agarose gel electrophoresis. The baseline of each reaction was equalized by calculating the mean value of the 5 lowest measured data points for each sample and subtracting this from each reading point. Background fluorescence was removed by setting a noise band. The number of cycles at which the best-fit line through the log-linear portion of each amplification curve intersects the noise band is inversely proportional to the log of copy number.

### Immunoprecipitation Assay

Cell extracts were incubated with 1 µg of anti-XIAP or -Smac antibody for 24 h at 4°C. The precipitates were further reacted with protein A-Sepharose beads (GE Healthcare/Amersham Biotech, Piscataway, NJ) and eluted by boiling for 5 min in Laemmli sample buffer. Electrophoretic separation of the immuno-precipitated proteins or cell lysates was done in 10% acrylamide gels, and bands were transferred onto Immobilon NC membranes (Millipore, Bedford, MA). For immuno-blotting, the membranes were probed with anti-XIAP, Smac, or caspase-3 antibody at 1/200 dilution. The blots were incubated with species-specific conjugated HRP secondary Abs (Jackson ImmunoResearch Laboratories) and signals revealed by ECL (GE Healthcare).

### Data Analysis

Each experiment was performed in duplicate and its average was used for quantification. Data are expressed as the mean ± SEM of averages from at least 3 experiments. ANOVA was used to assess the statistical significance of the differences, and a value of *p*<0.05 was considered statistically significant.

## Results

### NK Cells Enhance Radiosensitivity

**Figure 1:**
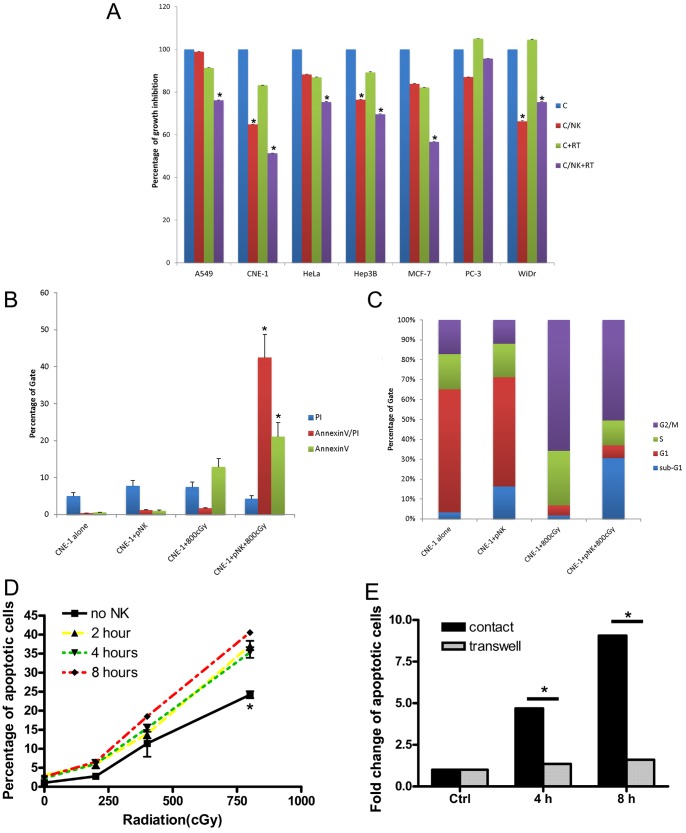
pNK and NK-92 cells sensitized tumor cells. 1×10^5^ of various tumor cells were seeded in 96-well tissue-culture plates, co-cultured with 2.5×10^5^ pNK cells for 4 h, washed and then exposed to 800 cGy of irradiation and evaluated 48 h late for cell proliferation by the MTS (A). C, cancer cell alone; C/NK, cancer cell and NK coculture; C+RT, cancer cell treated with 800 cGy radiation; C/NK+RT, cancer cell and pNK coculture followed by radiation. The apoptosis of CNE-1 cells after irradiation 48 h under various co-culture conditions that described previously was analyzed by (B) Annexin-V assay and (C) cell cycle analysis. (D) CNE-1 cells were incubated with NK-92 cells in 1∶2.5 ratios for 2, 4, and 8 h and irradiated at indicated doses. (E) 1×10^5^ CNE-1 cells were cultured in the lower chambers of transwells, and 2.5×10^5^ NK-92 cells were cultured in the upper chambers for 4 and 8 h. Both of (D) and (E) were assayed using Annexin-V to detect apoptotic cells (AnnexinV^+^). (*, *p*<0.05).

The ability of pNK cells to enhance radiation in cancer cell lines was examined by irradiating cells after a 4-h co-incubation with pNK. As shown in Fig. 1A, pNK cells were found to enhance radiation cytotoxicity in most cell lines in compared with medium alone control or radiation alone except PC-3 cell line. It seems to be a general phenomenon in rest cell lines. Since CNE-1 showed the most significant radiosensitization effect on pNK cells treatment (P<0.001), we choose CNE-1 cell for the following mechanistic study. Apoptotic efficacy of CNE-1 cells co-cultured with pNK cells was assessed by apoptosis assay and cell cycle analysis. Both of the percentage of late apoptotic cells (AnnexinV/PI) and early apoptotic cells (AnnexinV) were significantly increased as compared to the pNK control or the radiation treatment control (p<0.05) (Figure 1B) as well as sub-G1 ratio in cell cycle analysis (Figure 1C). Although CNE-1, Hep3B and WiDr showed significant growth inhibition by MTS assay after 4 h co-culture with NK cells (Figure 1A), the apoptosis assay did not reveal cell death (CNE-1, Figure 1 C and D; Hep3B and WiDr, Figure S1). The representative data for annexin-V and cell cycle analysis were showed in Figure S2A and B. Longer exposure to pNK before irradiation resulted in a greater effect of cytotoxicity significantly (p<0.05, Figure 1D). The decline in the radiation enhancing effect observed in cells co-cultured in transwells indicated a cell-cell contact-dependent mechanism (Figure 1E). These data suggest that a short, sublethal contact with NK cells increases the susceptibility of CNE-1 cells to radiation. Similar results for tumor cells cocultured with NK cell line (NK-92) was showed in Figure S2C and D. A dose response relationship that below 2.5∶1 of NK/tumor cells coculture resulted in less effect was found (Figure S3). Thus, we used NK-92 cell line to surrogate pNK cells and 2.5∶1 of NK/tumor cells ratio for the following experiments.

### NK-92 Contact Induced Extrinsic Pathway on CNE-1 Cells only Partially Related to NK-induced Radiation Enhancing Effect

**Figure 2:**
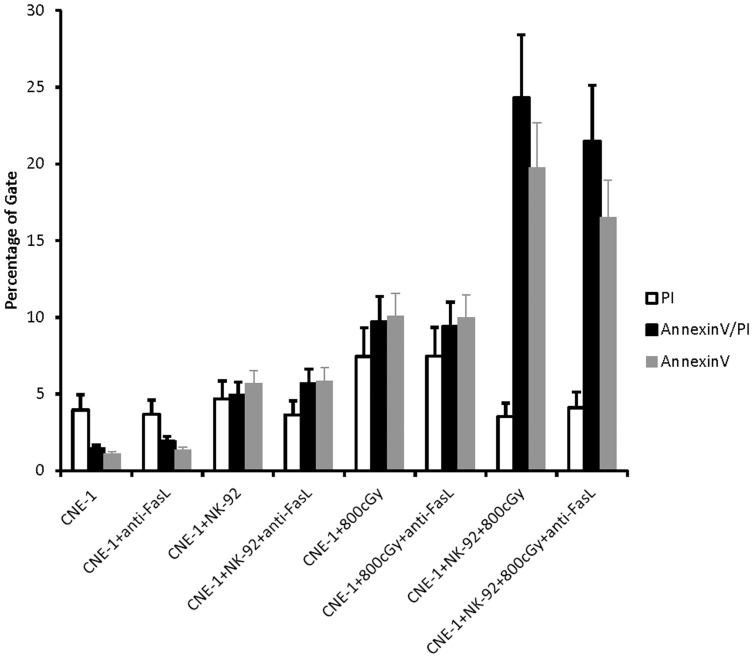
Effect of NK-92-treated CNE-1 cells on Fas blockage. CNE-1 cells were co-cultured with 2.5 fold NK-92 cells for 4 h in presence of anti-FasL blocking antibody (10 µg/ml). The percentage of apoptotic cells after irradiation 48 h under various co-culture conditions was analyzed by Annexin-V assay (AnnexinV^+^, D). Results from 3 independent experiments are shown; bars indicate mean ± SD.

NK-92 cell is known to trigger apoptotic signals via death receptor on target cells [24], whether death receptor activation plays the major role in NK cells radiosensitization effect is basically unknown. We found that Fas protein was significantly increased in CNE-1 cells after co-culture with NK-92 cells in a time and dose-dependent manner (Figure S4). Nevertheless, the radiation-enhancing effect was not significantly abolished by anti-FasL (Figure 2). A similar result was found in the TRAIL/DR5 system (data not shown). Both indicated death receptors were only partially involved in the radiosensitization effect of NK cells.

### Intrinsic Pathway of Apoptotic Protein Caspase-9 Involved in the NK-induced Radiation Enhancing Effect

**Figure 3:**
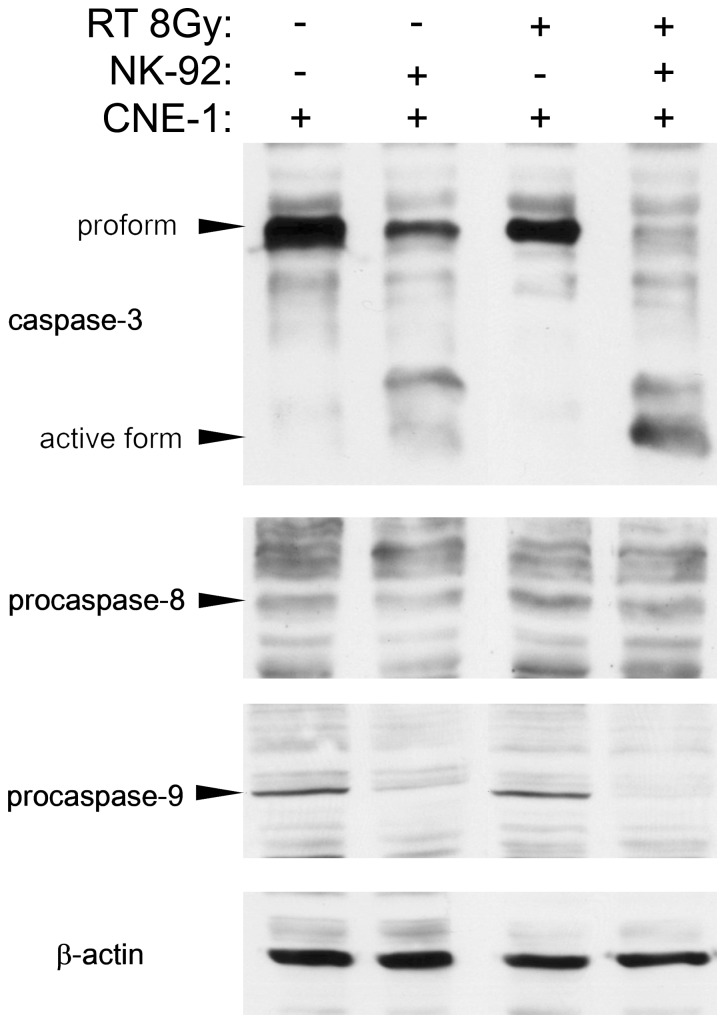
The caspase signaling pathway was induced after co-culture. CNE-1 cells were co-cultured with 2.5 fold of NK-92 cells for 4 h, then NK-92 cells were washed away, and CNE-1 cells were exposed to 800 cGy of radiation. Control cells of CNE-1 alone or CNE-1 cells that had been co-cultured were not irradiated. After 24 h of incubation, cells were harvested for western blot analysis of procaspase/caspase-3, procaspase-8, and procaspase-9 protein in lysates of CNE-1 alone (lane C), CNE-1 cells cultures with NK-92 cells (lane C/N). The arrows indicate cleaved (activated) caspase 3 at about 17 kDa and its precursor, pro-caspase 3, at about 43 kDa; precaspase 8, at about 55kDa; procaspase 9, at about 45kDa. β-actin was used as the internal control.

The effect of NK co-culture on caspase activation in CNE-1 cells with or without radiation was investigated next. Western blot analysis confirmed that procaspase-9 was cleavaged after NK cell co-culture (Figure 3). The cleavage of procaspase-9 indicated the activation of caspase-9 [25]. Caspase-3 activity was only slightly increased in the absence of radiation. On the other hand, caspase-8 was not activated in all treatments. Since caspase-9 is the primary caspase involved in the intrinsic mitochondrial pathway and caspase-8 is involved in the death receptor pathway, our results suggest that NK-92 may sensitize CNE-1 cells to radiation chiefly by activation of the intrinsic mitochondrial apoptosis pathway.

### Grz B Plays A Key Role in NK-92-induced Radiation Enhancing Effect

**Figure 4:**
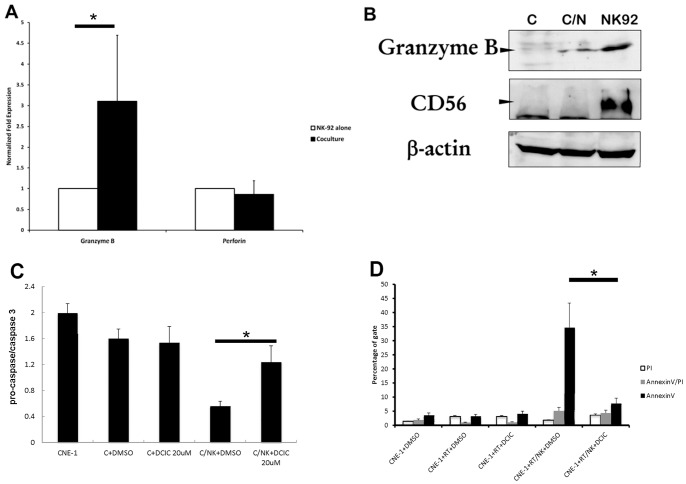
Granzyme B was secreted by NK-92 cells and penetrated into CNE-1 cells during co-culture. (A) Levels of Granzyme B and perforin mRNA expression in NK-92 cells co-cultured with or without CNE-1 cells for 4 h were measured by real-time RT-PCR. The amounts of mRNA are expressed relative to the amount of MBD-4 in each sample and are shown as the mean ± SD of 3 separate experiments. Significant differences in the expression in the presence or absence of the stimulators are indicated as * (*p*<0.05). (B) Quantitative analysis of Granzyme B protein in lysates of CNE-1 alone by western blotting (lane C); CNE-1 treated with NK-92 cells for 4 h then NK-92 cells removed (lane C/N); NK-92 cells that had been washed from CNE-1 cell co-culture (lane NK92). CD56 was used to demonstrate exclusion of contamination with NK-92 cells, and β-actin was used as the internal control. (C) DCIC pretreated with CNE-1 cells for 1 h, then co-cultured with 2.5 fold of NK92 cells for 4 h. After washing NK92 cells away, and CNE-1 cells were exposed to 800 cGy of radiation and analyzed for the ratio procaspase/caspase-3 by ImageJ (a decreased ratio is indicative of apoptosis). The bar chart was average of three independent experiments. (D) Annexin-V assay. (*, *p*<0.05).

Cytosolic Grz B is a major effector molecule of NK cells that initiates the proteolytic cascade inducing target cell death. The gene expression of Grz B, but not perforin, was increased in NK-92 cells after co-culture (Figure 4A). The release of Grz B into CNE-1 cells is evident in Figure 4B. As shown in Figure 4B, the level of Grz B in CNE-1 is very low (first line, top plant and Figure S5). After co-culture with NK cells, the level of Grz B was significantly increased (second line, middle plant) while radiation alone did not increase the Grz B level (Figure S5). The NK cell marker, CD56, was used to demonstrate the lack of contamination of NK-92 cells in the harvested CNE-1 cells after co-culture. Since Grz B play a role in the NK-92-induced radiation enhancing effect, DCIC, a Grz B inhibitor, was used to block the NK-92-induced signaling pathway. We found that 20 mM DCIC was the maximal dose that did not affect cell survival. This dose was used to inhibit the NK/radiation induced apoptosis by both procaspase/caspase 3 ratio and annexin-V assay (Figure 4C and D, p<0.05). These data further confirmed that Grz B was involved in the NK-92-induced radiation-enhancing effect.

### Radiation INDUCED SMAC to Counteract XIAP Binding of Caspase-3

**Figure 5:**
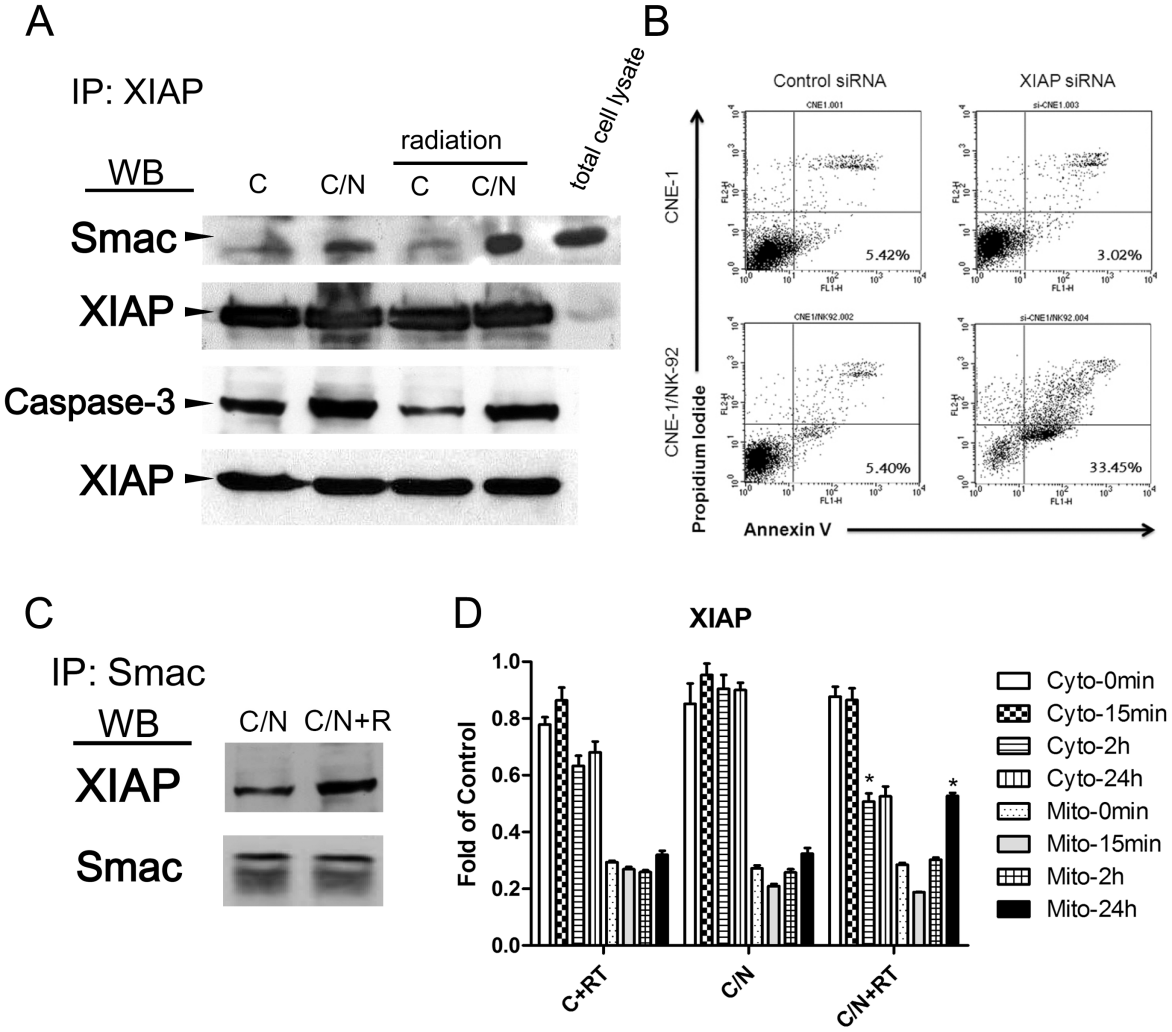
Caspase-3 was inhibited by XIAP and XIAP was downregulated by binding of Smac after radiation. CNE-1 cells (lane C) were treated with NK-92 cells for 4 h (lane C/N) before combined treatment with 800 cGy of radiation. (A) Cell lysates were immunoprecipitated with anti-XIAP antibody and immunoblotted with anti-Smac, anti-caspase-3 or anti-XIAP antibody. (B) CNE-1 cells were transfected with 80 nM of XIAP siRNA for 16 h and co-cultured with NK-92 cells for 4 h before NK-92 cells were washed away. The cells were assayed using Annexin-V to determine the percentage of apoptotic cells (AnnexinV^+^). (C) Cell lysates were immunoprecipitated by anti-Smac antibody and detected with anti-XIAP antibody by Western blot. (D) CNE-1 cells were treated with NK-92 cells for 4 h (C/N) before combined treatment with 800 cGy of radiation (C/N+RT) or CNE-1 treated with 800 cGy of radiation alone (C+RT). After treatment, cells were further incubated for 0 min, 15 min, 2 h, or 24 h, then harvested and fractionated into cytosolic (Cyto) and mitochondrial (Mito) fractions for assay by western blot. β-actin was used as the loading control for each fraction. Density of the XIAP normalized with β-actin was assayed by Image J. The bar chart was average of three independent experiments.

To address the question of how NK-92 cells activated the caspase pathway without completing the apoptotic process, we further investigated the changes of XIAP, an important anti-apoptotic molecule. Immunoprecipitated XIAP was exposed to caspase-3 and Smac (a proapoptotic protein); then, the binding efficiency was assessed by western blot. We tested whether radiation treatment facilitates the activation of the Smac to bind XIAP. Immunoprecipitation analysis showed that Smac binding to XIAP was markedly increased as compared with C/N without radiation (Figure 5A). After co-culture with NK-92 cells (C/N), XIAP binding of activated caspase-3 in CNE-1 cells increased without a change in the total amount of XIAP (Figure 5A). To confirm the key role of XIAP in resistance to NK-92-mediated killing, we specifically down-regulated XIAP by transfecting siRNA into CNE-1 cells. As shown in Figure 5B, knockdown of XIAP significantly enhanced NK-92-induced apoptosis. The finding was further confirmed by immunoprecipitation of Smac, and then incubating the precipitate with XIAP. XIAP/Smac complexes was increased in C/N plus radiation, as predicted (Figure 5C). A detailed time-dependent translocation of XIAP from the cytosol to mitochondria was investigated by analyzing mitochondrial and cytosolic fractions from 15 min to 24 h after radiation. A migration of XIAP from the cytosol to mitochondria was observed between 2 h and 24 h after RT (Figure 5D). The decreasing of XIAP in cytosol was significantly revealed at 2 h after RT, whereas increasing of XIAP in mitochondria was significantly shown at 24 h after RT. NK-92 cell treatment alone did not promote XIAP translocation (Figure 5D). The translocation of XIAP into mitochondria may initiate the release of Smac inside the mitochondria [26]. These data indicated that NK-92 co-culture with CNE-1 cells was insufficient to achieve apoptosis due to the induction of XIAP binding with caspase, while subsequent radiation released Smac into the cytosol to bind cytosolic XIAP, thus enabling apoptosis to proceed.

### pNK Cells Induced Radiation-enhancing Effect through XIAP

**Figure 6:**
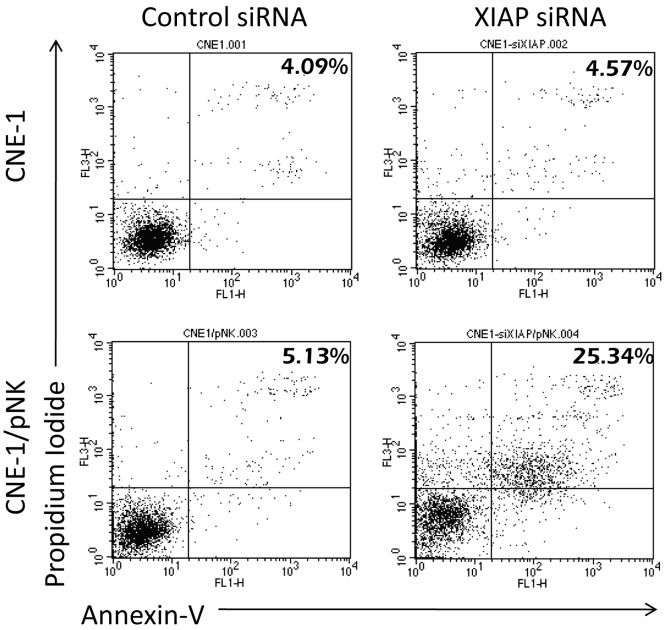
Primary NK cells sensitized tumor cells with same pathway. CNE-1 cells were transfected with 80 nM of XIAP siRNA for 16 h and co-cultured with pNK cells for 4 h before pNK cells were washed away. The cells were assayed using Annexin-V to determine the percentage of apoptotic cells.

**Figure 7:**
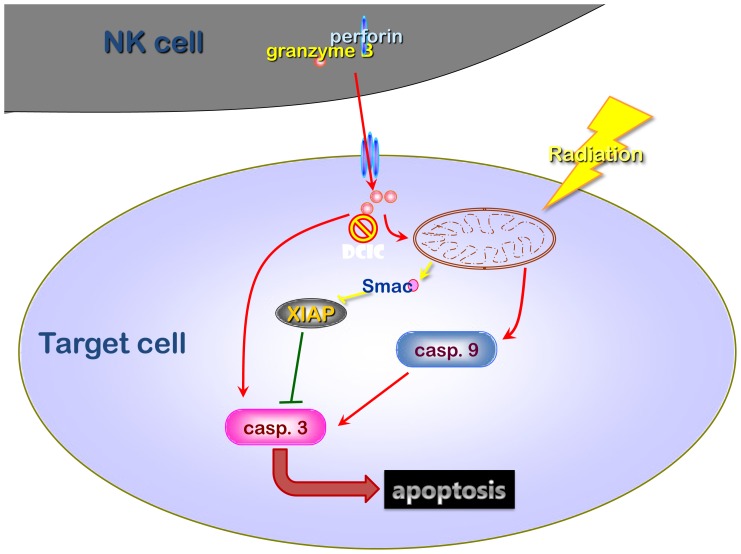
Mechanism of reciprocal interaction between NK cells and radiation in target cells. NK cells damage target cell through perforin/granzyme B and death receptor/caspase mediated pathway. The radiosensitisation effect through NK cell depends more on the perforin/granzyme B pathway. Without radiation, the suboptimal activation of NK cells cause up-regulation of XIAP. With radiation, the mitochondria releases Smac to neutralize XIAP and enhances NK cell-mediated cytotoxicity.

To further investigate whether the pNK cells could also sensitize CNE-1 cells to radiotherapy as well, we further knock-out XIAP by transfecting siRNA into CNE-1 cells. As shown in Figure 6, knockdown of XIAP also significantly enhanced pNK-induced apoptosis. These data indicated that both pNK and NK-92 shared similar mechanisms to induce radiosensitization effect. NK cells released granzyme B/perforin into target cells, triggered activation of the caspase pathway, and directly increased radiation-induced cell damage. The radiosensitising effect was significantly reduced when granzyme B was inhibited. If radiation was not administered, “the suboptimal activated” NK cells up-regulated of XIAP in target cells and thereby inhibited apoptosis. The adding of radiation significantly increased apoptosis by stimulating the release of mitochondrial Smac to neutralize the inhibitory effects of XIAP. Therefore, radiation is, in a broad sense, a Smac-inducing agent that may significantly increase the killing effect of NK cells. This model of reciprocal interaction between NK cells and radiation in target cells is illustrated in Figure 7.

## Discussion

Although the clinical benefits of combined immunotherapy and conventional therapy are well acknowledged, the underlying mechanisms involved in cell-cell contact level have not been well defined. Here, we show that NK cells and radiation could reciprocally help each other to induce tumor cell death. NK cells sensitize target cells to radiation by injection with Grz B upon cell-cell contact. Radiation induces the release of mitochondrial Smac to counteract the cytosolic XIAP self-protection mechanism and to enhance NK cell-mediated lysis.

Evidence in the literature suggests that combining vaccines and monoclonal antibody treatments with chemotherapy or radiotherapy has higher clinical response rates than individual treatment modalities [27], [28], [29]. Phase III clinical trials have shown the survival benefit [30]. Chemotherapy-treated tumor cells became sensitive to lysis by low-avidity cytotoxic T lymphocytes induced by specific immunotherapy [29]. Immune and tumor cell crosstalk must occur in the tumor microenvironment. We have tested seven tumor cell lines in NK/tumor coculture system, only PC-3 did not express significant radiosensitization effect after pre-incubation with NK cells. The reason for PC-3 did not respond to what we expect may due to PC-3 was more resistant to radiation according to previous publication [31]. Rudner et al. has found that PC-3 could be reversed to high levels of apoptotic cell death after radiation when Akt inhibitor was combined [31]. The induction of proapoptotic signals to certain cancer cells by NK/tumor cells coculture may depend on different experimental conditions in order to show optimal result. However, it should be a general phenomenon. In our study, we chose 2.5∶1 of NK/tumor cells ratio, this ratio is relatively high as compared to the in vivo tumor microenvironment. However, clinical results have long suggested the importance of healthy immune microenvironment but the mechanism remains unclear. Our result on NK cells just a small brick of whole picture of immune microenvironment.

NK cells are known to initiate tumor cell death, either by binding the death receptor CD95/Fas or by the release of granules containing perforin and the enzymatic molecule Grz B [32]. In spite of optimal cell engaging mechanisms, NK cell-mediated Grz B or death receptor signaling appears insufficient to induce the level of caspase-3 activity required to achieve target cell death without the help of radiation. Grz B fails to induce mitochondrial release of cytochrome c, and as a result, tumor cells escape from immune destruction.

A number of mechanisms were proposed to explain the failure of natural defense immunity [33], [34]. After serial analysis of the molecular mechanisms, we found that XIAP machinery is likely the most important defense mechanism. XIAP is the first well-characterized member of the inhibition of apoptosis protein family [35]. Knockdown of XIAP significantly enhanced the cytotoxic effect of NK92 cells. While a similar finding has been reported in cytotoxic T cells [36], we are the first group to propose that radiation may be regarded as a Smac-mediated agent to overcome immune resistance.

Radiation targets mitochondria and potentiates the effects of intermembrane space proteins, such as Smac. Both Smac and cytochrome c are co-released from mitochondria during UV-induced apoptosis, and this process is caspase-independent [37]. This release was believed to be triggered by the aggregation of Bax in the outer mitochondrial membrane to form a lipid-protein complex [37]. The release of additional apoptotic factors from the mitochondria, such as Smac, to inhibit XIAP activity, and to further activate caspase-9 to promote the cascade of activation events is necessary to complete the apoptotic pathway [36]. Our data provides evidence that Smac is a downstream effector molecule of radiation. Interestingly, Streceli *et al*. has reported that endogenous XIAP translocates from the cytosol to mitochondria to induce permeabilization of the outer membrane of mitochondria, leading to Smac release, which occurs early in the apoptosis process [26]. We agree with their observation that XIAP may switch the mitochondrial function from the anti- to pro-apoptotic form when radiation provides additional suicide signal to NK-contacted cells.

Apoptosis activation by NK-92 cells by the extrinsic pathway, which increased death receptor-mediated caspase 8, and the intrinsic pathway from Grz B, which induced caspase 9, is not sufficient to induce CNE-1 cell death. This phenomenon may present in most tumor microenvironment. Under such sublethal conditions, NK immunity is not sufficient to control tumor growth. The extrinsic or intrinsic apoptotic pathway from NK cell contact is not lethal without a concomitant mitochondrial release of Smac modality such as radiation proposed in this study. A reciprocal complementary relationship between NK cells and radiation on tumoricide is evident.

The concept of combined immunotherapy and radiotherapy has advanced recently [38]. Several studies have revealed that the quantitative measurement of tumor infiltrating lymphocytes in biopsy samples before chemotherapy or radiotherapy can be used as a predictor of the clinical effectiveness of treatment for cancer [39], [40]. Our findings provide a conceptual acceptable link for clinical implications on neoadjuvant immunotherapy. We found that NK cells that infiltrate tumors have potent radiation-enhancing effects in a sublethal dosage. Neoadjuvant immunotherapy aims to create a microenvironment favorable to cells of the innate immune system before radiotherapy is given. Theoretically, this is more effective than providing specific immunotherapy after radiotherapy, while ignoring the tumor microenvironment before radiation. We also found that radiation greatly enhanced the tumor susceptibility to immunologically mediated cell death, through the release of Smac. The dead cells, along with the danger signals delivered by irradiated tumor tissue, serve as links between the local response and the subsequent specific immune response [41]. Innate effector cells, including NK cells and dendritic cells in the tumor microenvironment, orchestrate the entire scenario [42].

In conclusion, we found that NK cells sensitize tumor cells to radiation and radiation sensitizes tumor cells to NK cell attack. Grz B transfer into tumor cells results in a radiation enhancing effect. Resistance of tumor cells to NK cell-mediated death is associated with endogenous XIAP inhibiting the caspase-3 triggered by immune cell attack. Radiation induced mitochondrial release of Smac to neutralize cytosolic XIAP. Therefore, the reciprocal complementation between NK cells and radiation to effect tumoricide is intriguing and important. A strategy of neoadjuvant immunotherapy to alter the immune milieu before radiotherapy is suggested.

## Supporting Information

Figure S1 **Apoptosis assay.** 1×10^5^ of (A) Hep3B cells and (B) WiDr cells were seeded in 96-well tissue-culture plates, co-cultured with 2.5×10^5^ pNK cells for 4 h, washed and then exposed to 800 cGy of irradiation and evaluated 48 h late for Annexin-V.
(PDF)

Figure S2 **The representative data for annexin-V and cell cycle analysis.** 1×10^5^ of CNE-1 cells were seeded in 96-well tissue-culture plates, co-cultured with 2.5×10^5^ pNK (A, B) or NK-92 (C, D) cells for 4 h, washed and then exposed to 800 cGy of irradiation and evaluated 48 h late for Annexin-V assay (A, C) and cell cycle analysis (B, D).
(PDF)

Figure S3 **Dosage analysis on NK/tumor cells ratio.** CNE-1 cells were seeded into 6-well plates and co-cultured with NK-92 cells at the indicated ratios for 4 h. The apoptotic cells were measured by Annexin-V assay.
(PDF)

Figure S4 **CNE-1 expressed Fas after co-culture with NK-92 cells.** The expression of Fas was measured by flow cytometry. CNE-1 cells were seeded into 6-well plates and co-cultured with 2.5 fold NK-92 cells at the indicated times (A). CNE-1 cells were co-cultured with NK-92 cells at the indicated ratios for 4 h (B).
(PDF)

Figure S5 **Granzyme B expression assay.** Granzyme B protein in lysates of CNE-1 alone by western blotting (lane C); CNE-1 treated with 800 cGy of irradiation (lane C/RT); lysates of NK-92 cells (lane NK92). β-actin was used as the internal control.
(PDF)
