# Supplementary figures and images for: Reciprocal Complementation of the Tumoricidal Effects of Radiation and Natural Killer Cells
Source: PLoS One. 2013 Apr 25;8(4):e61797. doi: 10.1371/journal.pone.0061797 (PMC3636248; doi:10.1371/journal.pone.0061797)

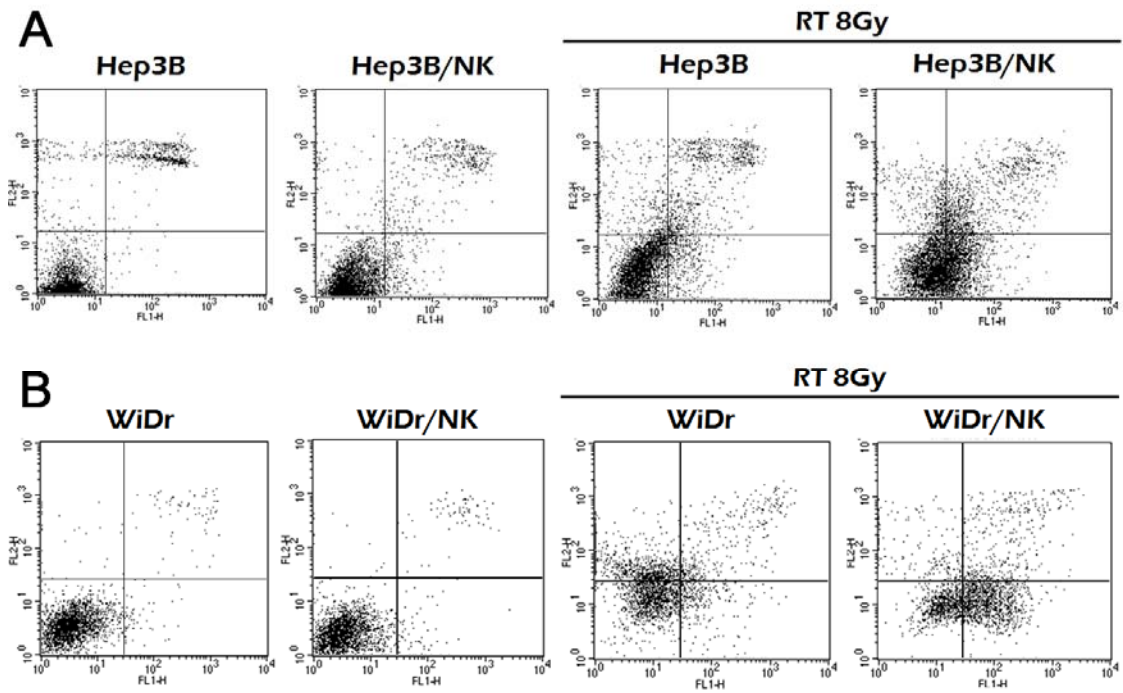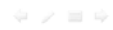

**Figure S1:** Apoptosis assay.

Supplement: Figure S1 — Apoptosis assay. 1×10^5^ of (A) Hep3B cells and (B) WiDr cells were seeded in 96-well tissue-culture plates, co-cultured with 2.5×10^5^ pNK cells for 4 h, washed and then exposed to 800 cGy of irradiation and evaluated 48 h late for Annexin-V. (PDF) [file pone.0061797.s001.pdf]

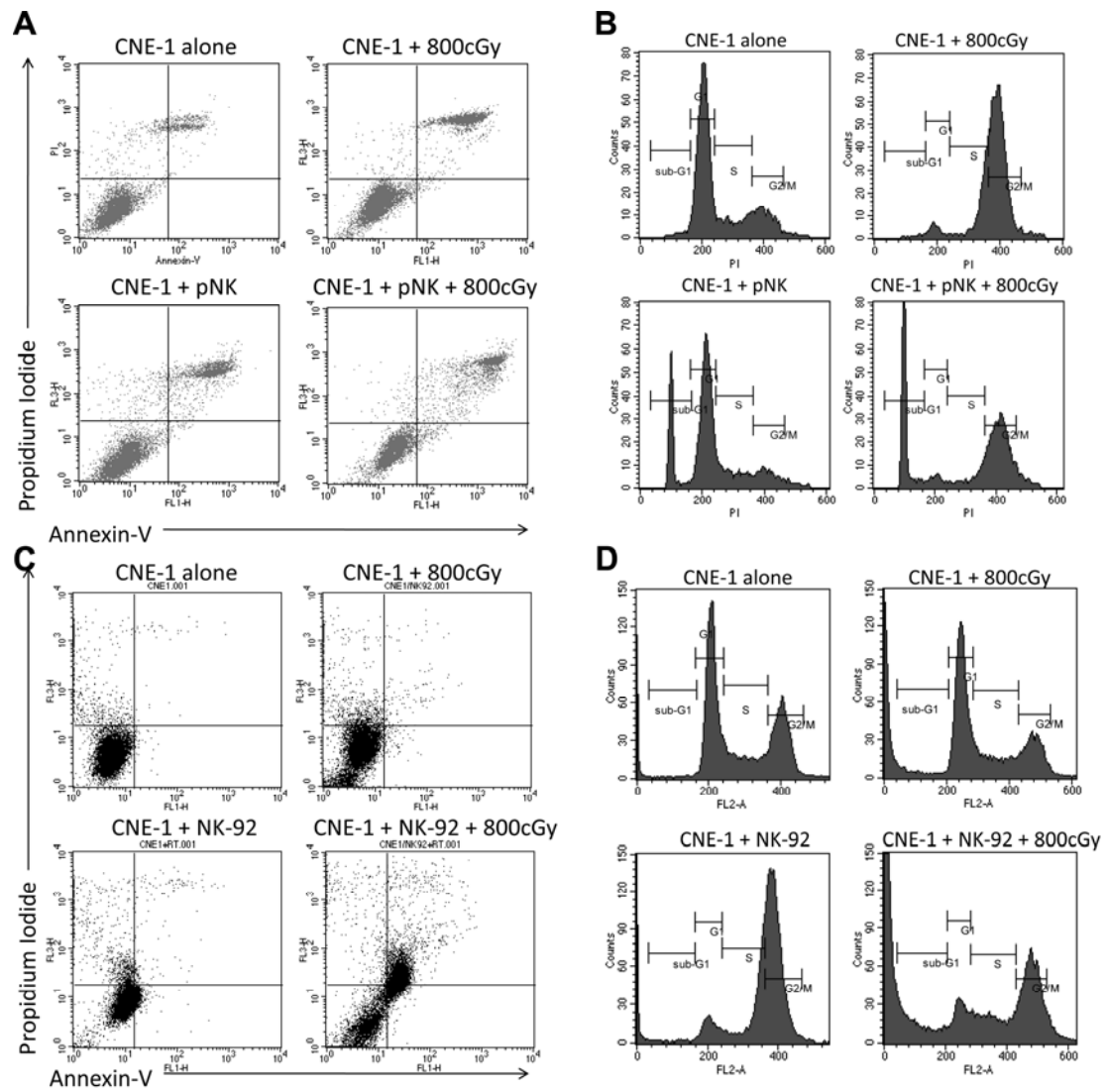

**Figure S2:** The representative data for annexin-V and cell cycle analysis.

Supplement: Figure S2 — The representative data for annexin-V and cell cycle analysis. 1×10^5^ of CNE-1 cells were seeded in 96-well tissue-culture plates, co-cultured with 2.5×10^5^ pNK (A, B) or NK-92 (C, D) cells for 4 h, washed and then exposed to 800 cGy of irradiation and evaluated 48 h late for Annexin-V assay (A, C) and cell cycle analysis (B, D). (PDF) [file pone.0061797.s002.pdf]

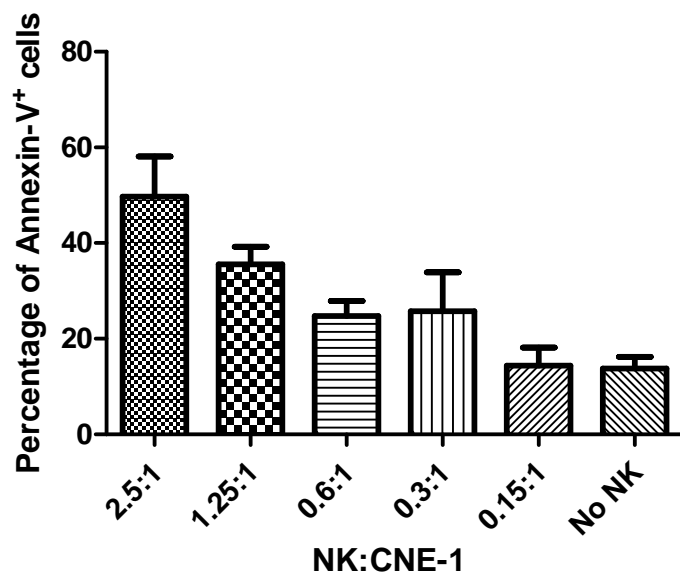

**Figure S3.** Dosage analysis on NK/tumor cells ratio.

Supplement: Figure S3 — Dosage analysis on NK/tumor cells ratio. CNE-1 cells were seeded into 6-well plates and co-cultured with NK-92 cells at the indicated ratios for 4 h. The apoptotic cells were measured by Annexin-V assay. (PDF) [file pone.0061797.s003.pdf]

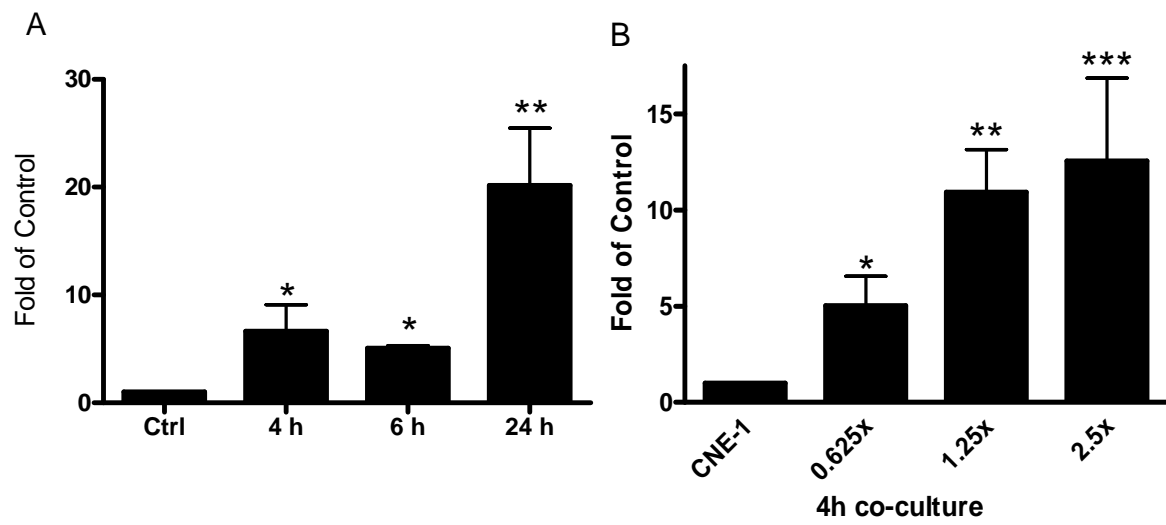

**Figure S4.** CNE-1 expressed Fas after co-culture with NK-92 cells.

Supplement: Figure S4 — CNE-1 expressed Fas after co-culture with NK-92 cells. The expression of Fas was measured by flow cytometry. CNE-1 cells were seeded into 6-well plates and co-cultured with 2.5 fold NK-92 cells at the indicated times (A). CNE-1 cells were co-cultured with NK-92 cells at the indicated ratios for 4 h (B). (PDF) [file pone.0061797.s004.pdf]

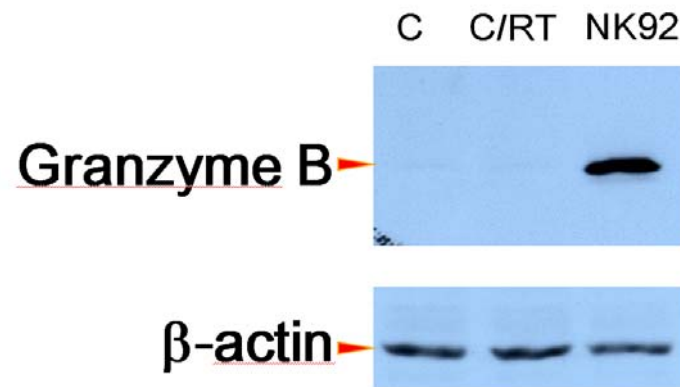

**Figure S5.** Granzyme B expression assay.

Supplement: Figure S5 — Granzyme B expression assay. Granzyme B protein in lysates of CNE-1 alone by western blotting (lane C); CNE-1 treated with 800 cGy of irradiation (lane C/RT); lysates of NK-92 cells (lane NK92). β-actin was used as the internal control. (PDF) [file pone.0061797.s005.pdf]
